# High Expression of Cancer-IgG Is Associated With Poor Prognosis and Radioresistance *via* PI3K/AKT/DNA-PKcs Pathway Regulation in Lung Adenocarcinoma

**DOI:** 10.3389/fonc.2021.675397

**Published:** 2021-06-02

**Authors:** Xiongtao Yang, Guohui Wang, Jing You, Runchuan Gu, Xiaohong Xu, Changdan Xu, Hao Wang, Ruisong Zhao, Xiaoyan Qiu, Guangying Zhu

**Affiliations:** ^1^ Department of Radiation Oncology, Peking University China-Japan Friendship School of Clinical Medicine, Beijing, China; ^2^ Department of Radiotherapy, Second Hospital of Hebei Medical University, Shijiazhuang, China; ^3^ Key Laboratory of Carcinogenesis and Translational Research (Ministry of Education), Department of Radiation Oncology, Peking University Cancer Hospital and Institute, Beijing, China; ^4^ Department of Radiation Oncology, China-Japan Friendship Institute of Clinical Medicine, Beijing, China; ^5^ Department of Radiation Oncology, Center of Respiratory Medicine, China-Japan Friendship Hospital, Beijing, China; ^6^ Institute of Respiratory Medicine, Chinese Academy of Medical Sciences, Beijing, China; ^7^ Department of Radiation Oncology, National Clinical Research Center for Respiratory Diseases, Beijing, China; ^8^ Department of Radiation Oncology, World Health Organization (WHO) Collaborating Centre for Tobacco Cessation and Respiratory Diseases Prevention, Beijing, China; ^9^ Department of Pathology, China-Japan Friendship Hospital, Beijing, China; ^10^ Department of Immunology, School of Basic Medical Sciences, Peking University, Beijing, China; ^11^ Peking University Center for Human Disease Genomics, Beijing, China

**Keywords:** cancer-IgG, lung adenocarcinoma, radioresistance, PI3K/AKT/DNA-PKcs pathway, RP215

## Abstract

**Background:**

Lung adenocarcinoma (LUAD) is the dominant type of lung neoplasms, and radiotherapy is its mainstay treatment, yet poor prognosis caused by radioresistance remains problematic. Cancer-derived immunoglobulin G (cancer-IgG) has been detected in multiple cancers and plays important roles in carcinogenesis. This study aimed to demonstrate that cancer-IgG is associated with poor prognosis of LUAD and to identify its role in radioresistance.

**Methods:**

Cancer-IgG expression was detected by immunohistochemistry from 56 patients with stage III LUAD and by western blot and immunofluorescence in LUAD cell lines and in a human bronchial epithelial cell line. The effects of cancer-IgG silencing on the proliferation and apoptosis of PC9 and H292 cells were evaluated by plate cloning and apoptosis assay; the effects of cancer-IgG silencing on DNA damage repair ability and radiosensitivity were evaluated by colony-forming assay, γH2AX immunofluorescence, and neutral comet assay. Finally, we used the protein phosphorylation microarray and western blot to explore mechanisms involving cancer-IgG that increased radioresistance.

**Results:**

Cancer-IgG is widely expressed in stage III LUAD, and the overall survival and disease-free survival of patients with positive expression are notably lower than those of patients with negative expression, indicating the associations between cancer-IgG and poor prognosis as well as radioresistance. The expression of cancer-IgG in the four LUAD cell lines was located mainly on the cell membrane and cytoplasm and not in the normal lung epithelial cell. Knockdown of cancer-IgG in PC9 and H292 cells resulted in increased apoptosis and negatively affected cancer cell proliferation. After irradiation, silencing of cancer-IgG showed a decrease in colonies as well as increases in the Olive tail moment and γH2AX foci in nucleus, indicating that the knockdown of cancer-IgG resulted in a decrease in the damage repair ability of DNA double-strand breaks in LUAD cells and an enhanced radiosensitivity. The expression of p-AKT, p-GSK3β, and p-DNA-PKcs decreased in the knockdown group after radiotherapy, suggesting that cancer-IgG could affect radiotherapy resistance by mediating double-strand breaks damage repair in LUAD cells through the PI3K/AKT/DNA-PKcs pathway.

**Conclusions:**

This study revealed that cancer-IgG regulates PI3K/AKT/DNA-PKcs signaling pathways to affect radioresistance of LUAD and associated with poor prognosis.

## Introduction

Lung cancer represents the highest cancer incidence and remains the leading cause of cancer mortality worldwide (1). More than 85% of patients with lung cancer are pathologically diagnosed as having non-small cell lung cancer (NSCLC); accounting for approximately 50% of NSCLC, lung adenocarcinoma (LUAD) is the most commonly histological subtype of NSCLC (2). The clinical application of radiation for cancer therapeutic purposes began when x-ray was discovered by a German physicist, Roentgen, in 1895, and radiation has now become one of the major approaches of malignant tumor treatment after centuries of technological and equipment-related progression (3). Many large-scale clinical studies conducted in recent years involving patients with NSCLC (including LUAD) confirmed the survival time and quality-of-life improvements gained as a result of radiotherapy, and these results consequently contributed to the definition of radiotherapy as a standard therapy for NSCLC (4–6). However, local recurrence, as well as poor prognosis of patients resulting from radioresistance observed in tumor cells, has now become a major factor restricting the efficacy and additional development of radiotherapy; the 5-year survival rate of NSCLC remains less than 30% (7). Consequently, clarification about the mechanism of radioresistance in LUAD is urgently needed, and a novel theory of radiosensitization must be established to improve the survival of patients.

Qiu confirmed that epithelial tumors can produce immunoglobulin G and so established a new field of research into cancer-derived immunoglobulin G (cancer-IgG) (8). Cancer-IgG has been confirmed in a variety of tumors and is associated with poor prognosis (9–14). The discovery of the monoclonal antibody RP215, which specifically recognizes epitopes in the cancer-IgG heavy chain, has accelerated research about cancer-IgG (15). RP215-recognizable cancer-IgG interacts with integrin α6β4 to promote oncogenic activities of lung squamous cell carcinomas *via* activation of FAK and Src signaling (11). A recent study found that cancer-IgG can be secreted into the tumor microenvironment and can bind to sialic acid–binding immunoglobulin-type lectins of CD4+ and CD8+ effector T cells to directly inhibit the proliferation of CD4+ and CD8+ T cells and significantly promote tumor growth (16). Interestingly, our studies demonstrated that cancer-IgG can affect the radiosensitivity of tumors; we hypothesized that cancer-IgG can participate in irradiation-induced DNA double-strand breaks (DSBs) repair, which is considered a critical factor for LUAD radioresistance.

Several mechanisms of radioresistance involving cancer stem cells, apoptosis, reactive oxygen species, and DNA damage repair have been reported (17). DSBs caused by radiation in tumor cells are mainly repaired *via* nonhomologous end joining (NHEJ) and homologous recombination pathways, and repair by NHEJ is dominant throughout the cell cycle (18). DNA-dependent protein kinase (DNA-PK) is a key participant in DNA damage response and is instrumental in the NHEJ pathway, which serves to detect and repair DSBs. Inhibition of DNA-PK has resulted in an observable increase in tumor radiosensitivity (19). As a key complement of DNA-PK, phosphorylation of DNA-PK catalytic subunit (DNA-PKcs) is regulated by the PI3K/AKT pathway, which suggests that specific inhibition of DNA-PKcs–dependent DSBs repair *via* an AKT target can lead to an increase in the radiosensitivity of tumor cells (20). Gao et al. (21) demonstrated that bevacizumab directly inhibits the phosphorylation of VEGR2/PI3K/AKT/DNA-PKcs signaling components in endothelial cells induced by irradiation to inhibit endothelial DSB repair and increases the radiosensitivity of xenograft tumors in NSCLC. However, the effect of cancer-IgG on the radioresistance of LUAD cells has not been determined. In this study, we aimed to demonstrate that cancer-IgG is associated with the prognosis of patients with LUAD and examine its role in radioresistance.

## Material and Methods

### Collection and Processing of Clinical Specimens

The tumor tissue of 56 patients with LUAD admitted to the Peking University Cancer Hospital from October 2008 to January 2017 were collected. Their basic information was recorded, including age, gender, cigarette smoking history, disease stage, T classification, N classification, primary lesion diameter, visceral pleura invasion, vascular tumor thrombus, cancer-IgG expression, and disease-free survival (DFS), which was defined as the time from radical operation to disease recurrence. Patients who were alive without recurrence or lost to follow-up had their data censored at last available assessment. Patients who died from other causes without prior recurrence had their data censored at the date of death. Overall survival (OS) was defined as the date from preparation for treatment to the date of death from any cause or to the time of the last follow-up. All patients with LUAD had undergone radical operation and postoperative radiotherapy plus four cycles of chemotherapy; the radiation dose was 50 Gy in 25 fractions to the clinical target volume. All procedures were approved by the institutional review board.

### Cell Culture and Transfection

The human tracheal epithelial cell line (BEAS-2B) and the LUAD cell lines H292 and A549 were purchased from the National Biomedical Laboratory Cell Resource Bank. The LUAD cell lines H1299 and PC9 were obtained from the radiation department of the Peking University Cancer Hospital (Beijing, China). The H292, PC9, and H1299 lines were cultured in RPMI-1640 (Hyclone) with 10% fetal bovine serum (FBS) (Gibco), whereas the BEAS-2B and A549 cell lines were cultured in DMEM (Hyclone) with 10% FBS at 37°C in a humidified atmosphere of 95% O_2_ and 5% CO_2_. SiRNAs against the constant region of the Ig γ−chain (siRNA1, 5′−GGU GGA CAA GAC AGU UGAG−3′; and siRNA2, 5′−AGU GCA AGG UCU CCA ACAA−3′) and the non-silencing control RNA (scramble, 5′−UUC UCC GAA CGU GUC ACGU−3′) were produced by Suzhou GenePharma Co., Ltd. The siRNAs and scramble-RNA were transfected into the PC9 and H292 lines for 48 hours using lipofectamine 3000 (Thermo Fisher Scientific) and then were harvested according to the manufacturer’s protocol.

The pGCSIL-GFP- target short hairpin RNA (shRNA) lentiviral vectors (pGCSIL is a lentiviral vector) were purchased from Shanghai GeneChem Co., Ltd, and the shRNA sequences were the same as the siRNA2 sequences (5′−AGU GCA AGG UCU CCA ACAA−3′); the scramble shRNA sequences were the same as the non-silencing control RNA sequence (5′−UUC UCC GAA CGU GUC ACGU−3′). The shRNAs were transferred with polybrene (10 mg/mL; Sigma) into PC9 and H292 cells according to the shRNA product manual. Stable cells were screened with GFP fluorescence and puromycin (2 µg/mL). The knockdown efficiency of cancer-IgG was verified by western blot.

### Immunohistochemistry and Scoring

RP215 was provided by professor Xiaoyan Qiu of Peking University. We performed the experiment according to the procedure previously described for immunohistochemistry staining and cancer-IgG staining scores calculation (22). The score for RP215 staining was described as negative when the score was 0 to 3 and as positive when the score was 4 to 12.

### Immunofluorescence

Cells were grown on coverslips in four-well slides (Millicell EZ). The coverslips were fixed with 95% ethanol and permeabilized in 0.1% Triton X-100. Cells were blocked goat serum (ZLI-9056; ZSGB-Bio) and then incubated with RP215 or γH2AX (#80312; CST) antibody. After incubation with a secondary antibody for 1 h, the samples were counterstained with DAPI and imaged by a confocal microscope (Leica, Germany).

### Irradiation

PC9 and H292 cells were selected for radiation in the logarithmic growth phase, with the following x-ray irradiator (Varian) parameters: 6 MV x-ray energy and a dose rate of 4 Gy/min. After setting the dose according to the purpose, we placed the cell culture dish on a horizontal bed at a distance of 100 cm from the radiation source to the cell and covered it with a 1.5-cm-thick imitation human tissue pad. According to the International Atomic Energy Agency TRS-398 Code of Practice, a calibrated ionization chamber dosimeter was used for radiation dose verification.

### Cell Proliferation Assay

PC9 and H292 cells transfected with the siRNAs and scramble-RNA were cultured in six-well plates with approximately 300 cells/well and were incubated for 14 days. We discarded the medium in the six-well plates and washed with phosphate-buffered saline solution three times, adding 2 mL of 4% paraformaldehyde to each well and fixing them for 15 min at room temperature. Then, we discarded the 4% paraformaldehyde and added 2 mL of crystal violet working solution (DZ0056; Leagene) to each well for staining at room temperature for 30 min, slowly washing away the excess staining solution with running water and drying at room temperature. A colony was considered surviving when 50 or more cells were counted.

### Cell Apoptosis: Flow Cytometry Analysis

PC9 and H292 cells transfected with the siRNAs and scramble-RNA were cultured in six-well plates and incubated for 24 h; the cells were harvested and then incubated with FITC-annexin V and 7-AAD/PI staining buffer (KGA1030-50; KeyGen Biotech) at room temperature for 15 min. Cell apoptosis was measured with a FACS Calibur flow cytometer (BD Biosciences). Data analysis was performed with FlowJo software (Tree Star). FITC-annexin V– and PI-negative cells were considered viable; FITC-annexin V–positive and PI-negative cells represented early apoptosis cells; and FITC-annexin V– and PI-positive cells corresponded to late apoptosis or already dead cells.

### Colony-Forming Assay

After spreading the cells in a six-well plate for 24 hours, irradiate them with 0, 2, 4, 6 or 8 Gy. The subsequent steps matched those of section 2.6. The formula for the cell colony-forming rate and the cell survival score of each group is as follows: colony-forming rate plating efficiency (PE) = number of clones formed by cells after irradiation ÷ number of inoculated cells in this group. The cell survival fraction = PE of a certain dose irradiation group ÷ PE of the group with 0-Gy irradiation. The survival curve was graphed according to the survival fraction and dose in GraphPad Prism software.

### Neutral Comet Assay

This assay was carried out using a Trevigen kit according to the manufacturer’s instructions. Briefly, PC9 and H292 cells were resuspended and collected after 3 h of 2/4-Gy irradiation. The concentration of 2×10^5^/mL cell suspension and low-melting-point agarose were mixed evenly according to the volume ratio of 1:10, and then 50 µL of the mixture was dropped into the slice hole. The solidified slices with neutral electrophoresis buffer were electrophoresis at 21V voltage for 45 min and then were dried and stained with PI. Take the image under the fluorescence microscope, download the Comet Assay Software Project (CASP) to analyze the Olive tail moment of the cell, select the comet image to be analyzed with the check box, click the analysis function key and save. The main curve of the analysis curve is unimodal, and if it is bimodal, apoptotic cells are excluded. The distribution of DNA migration distance (comet tail length) and DNA content (fluorescence intensity) are linearly related to the degree of DNA damage. Olive tail moment is defined as: the product of tail DNA content and tail length, which is the main index to evaluate the degree of DNA damage in a single cell.

### Protein Phosphorylation Microarray Analysis

The Phospho Explorer Antibody Microarray was conducted by Full Moon BioSystems (Sunnyvale, CA). Whole-cell lysates from H292-sh-scramble and H292-sh-cancer-IgG treated with 6-Gy irradiation were harvested using a protein extraction buffer (Full Moon BioSystems). The protein microarray experiment was performed by Wayen Biotechnology (Shanghai, China) according to their established protocol. The fluorescence signal of each antibody was obtained from the fluorescence intensity of this antibody spot. A ratio computation was used to measure the extent of protein phosphorylation. The phosphorylation ratio was calculated as follows: phosphorylation ratio = phospho value ÷ unphospho value.

### Western Blot

Whole-cell lysates were prepared using a RIPA lysis buffer, supplemented with complete EDTA-free protease inhibitor mixtures and phosphatase inhibitors (Roche Diagnostics). Protein samples were separated on SDS-polyacrylamide gels. For western blot, separated proteins were transferred into PVDF membranes (Thermo Fisher). The membranes were incubated overnight at 4°C with primary antibodies and then incubated with a corresponding secondary antibody for 1 h at room temperature, when membranes were detected with an enhanced chemiluminescence reaction kit (Thermo Scientific Pierce). The primary antibodies were as follows: DNA-PKcs (ab102970, Abcam), p-DNA-PKcs (ab103970, Abcam), AKT (#4691, CST), p-AKT (#4060, CST), GSK3β (#12456, CST), p-GSK3β (#9323, CST), RP215, and GAPDH (HX1828, HuaxingBio).

### Statistical Analyses

The Kaplan-Meier method was used to analyze the correlation between cancer-IgG and OS or DFS. The survival curves were compared using a log-rank test. Univariate and multivariate Cox regression analysis evaluated the cancer-IgG significantly related to OS or DFS according to *P* values < 0.05. Cancer-IgG and clinicopathological features was carried out with a χ2 test. Data were presented as mean ± standard deviation. Student’s t test was used for the comparison between two groups. *P* values < 0.05 were considered statistically significant. All statistical evaluations were performed with SPSS version. 20.0.

## Results

### Cancer-IgG Had High Expression in LUAD and Was Associated With Poor Prognosis

**Figure 1:**
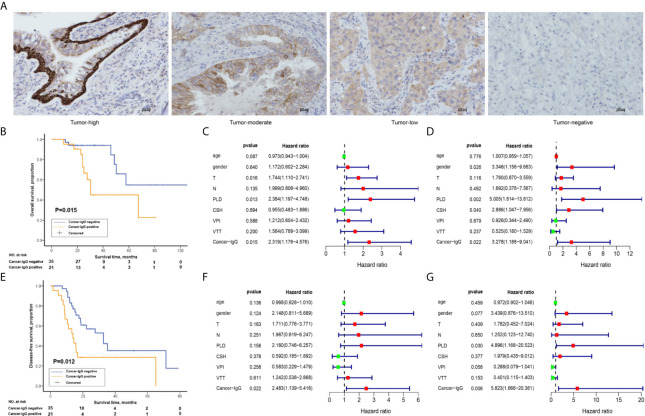
The relationship between the expression of cancer-derived immunoglobulin G (cancer-IgG) and survival outcome in 56 patients with stage III lung adenocarcinoma (LUAD). **(A)** Different expression levels of cancer-IgG in LUAD tissue sections. Scale bar, 100 μm. **(B)** Kaplan-Meier analysis of the overall survival (OS) between positive and negative cancer-IgG expression groups. **(C, D)** Univariate and multivariate analyses using a Cox proportional hazards model for the OS of patients with LUAD. **(E)** Kaplan-Meier analysis of the disease-free survival (DFS) between positive and negative cancer-IgG expression groups. **(F, G)** Univariate and multivariate analyses using a Cox proportional hazards model for the DFS of patients with LUAD. Age; gender: 0 (female), 1 (male); T: 1, 2, 3, 4; N:1, 2, 3; primary lesion diameter (PLD): 0 (< 4 cm), 1 (≥ 4 cm or double lesions); cigarette smoking history (CSH): 0 (never), 1 (former or current); visceral pleura invasion (VPI): 0 (no), 1 (yes); vascular tumor thrombus (VTT): 0 (no), 1 (yes); cancer-IgG: 0 (negative expression), 1 (positive expression).

Clinical data and pathological slides were collected from 56 patients with stage III LUAD for analysis of cancer-IgG expression by immunohistochemistry staining; 21 were positive and 35 were negative for cancer-IgG. Cancer-IgG is mostly located in the endoplasm and cell membrane (**Figure 1A**). The relationship between cancer-IgG expression and pathological characteristics of LUAD was analyzed (**Table 1**). Results showed that the expression of cancer-IgG was approximately correlated with N classification (*P* = 0.093) and vascular tumor thrombus (*P* = 0.069), suggesting that cancer-IgG may correlate with tumor metastasis. Such a conclusion matched that of our past analysis of cancer-IgG expression in NSCLC that involved data mining and tissue microarray (23).

**Table 1:** Association between cancer-IgG expression and clinicopathological features of patients with lung adenocarcinoma.

Characteristics	No. (%) of cases	Cancer-IgG		*P*
		Negative	Positive	
Age (years)				1.000
≥60	24 (42.9)	15	19	
<60	32 (57.1)	20	12	
Gender				0.367
Male	25 (44.6)	14	11	
Female	31 (55.4)	21	10	
Disease stage				0.659
IIIA	50 (89.3)	30	20	
IIIB	4 (7.1)	2	1	
T classification				0.594
1	22 (39.3)	15	7	
2	31 (55.3)	19	12	
3	2 (3.6)	1	1	
4	1 (1.8)	0	1	
N classification				0.093
1	4 (7.1)	4	0	
2	49 (87.5)	28	21	
3	3 (5.4)	3	0	
Primary lesion diameter (cm)				0.708
<4	39 (69.6)	25	14	
≥4 or double lesions	17 (30.4)	10	7	
Cigarette smoking history				0.664
Never	33 (58.9)	22	11	
Former	8 (14.3)	4	4	
Current	15 (26.8)	9	6	
Visceral pleura invasion				0.797
No	22 (39.3)	15	7	
Yes	32 (57.1)	19	13	
NA	2 (3.6)	1	1	
Vascular tumor thrombus				0.069
No	32 (57.1)	24	8	
Yes	16 (28.6)	8	8	
NA	8 (14.3)	3	5	

Cancer-IgG, cancer-derived immunoglobulin G; N, node; NA, not available; T, tumor.

Studies on cancer-IgG expression level and the survival prognosis for patients have shown that cancer-IgG expression significantly correlates with survival time. Kaplan-Meier analysis (*P* = 0.015, log-rank test; **Figure 1B**) and univariate/multivariate Cox regression analyses (**Figures 1C, D**) have shown that patients with positive cancer-IgG expression have poor prognoses and that positive expression of cancer-IgG could serve as an independent risk factor for OS. DFS refers to the period of time from radical operation to progress of disease, highlighting the efficacy of adjuvant radiotherapy and chemotherapy. The significant difference (*P* = 0.012, log-rank test; **Figure 1E**) of DFS among patients with different cancer-IgG expressions suggests a potential association between cancer-IgG expression and radioresistance. A significant difference in DFS between expression levels was found through univariate/multivariate Cox regression analyses (**Figures 1F, G**), indicating that cancer-IgG could be an independent risk factor for the prognosis of patients with stage III LUAD and could be associated with radiosensitivity.

### Cancer-IgG Knockdown Suppresses LUAD Progression

**Figure 2:**
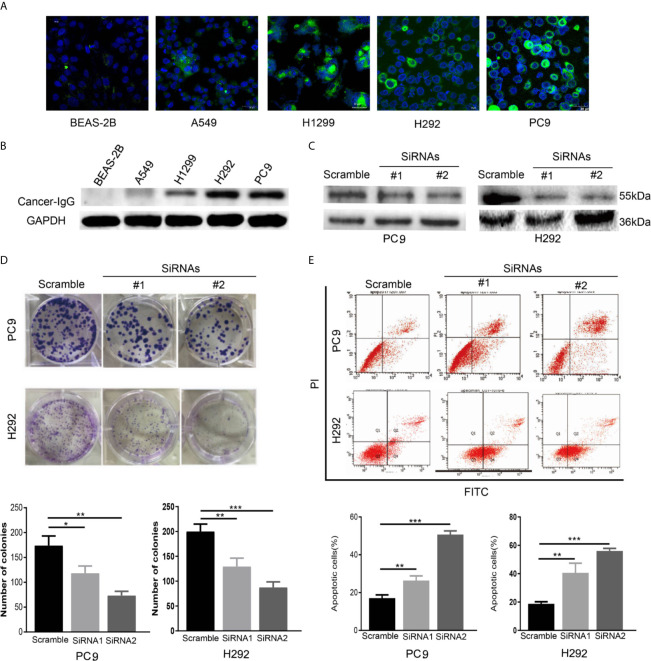
The expression and function of cancer-IgG in LUAD cell lines and normal lung epithelial cells. **(A, B)** Immunofluorescence staining and western blot analysis are used to show cancer-IgG expression in a normal lung–derived cell line (BEAS-2B) and four LUAD cell lines (PC9, H292, H1299, A549). Scale bar, 30 μm. **(C)** Western blot is used for testing the efficiency knockdown of cancer-IgG in PC9 and H292. **(D)** Plate cloning assay to evaluate the effect of silencing cancer-IgG on the proliferation of PC9 and H292 cells (n=3). **(E)** Flow cytometry assay to evaluate the effect of silencing cancer-IgG on the apoptotic ability of PC9 and H292 cells (n=3). *p < 0.05; **p < 0.01; ***p < 0.001.

Many studies have explored the expression of cancer-IgG in lung cancer (11, 12, 23). We observed the expression and location of cancer-IgG in a variety of LUAD cell lines and in normal lung epithelial cells (**Figures 2A, B**), confirming the existence of cancer-IgG in multiple LUAD cell lines (highest in PC9 and H292 and negative in BEAS-2B), mainly located in the endoplasm and cell membrane (consistent with previous immunohistochemistry results). By constructing siRNA that targets the *IGHG1* (immunoglobulin heavy constant gamma 1) gene, we performed cancer-IgG knockdown within PC9 and H292 cells; siRNA2 presented to be more efficient (**Figure 2C**).

A plating cloning assay was performed to evaluate cell viability and identified significant inhibition of PC9 and H292 cell proliferation as the expression of cancer-IgG was suppressed (**Figure 2D**). According to the result of a flow cyto-apoptosis assay, the ratio of cells undergoing apoptosis after cancer-IgG knockdown increased significantly, which indicated that cancer-IgG knockdown significantly promoted apoptosis of PC9 and H292 cells (**Figure 2E**). Taken together, our results suggest that reduced cancer-IgG expression attenuates LUAD cell proliferation and promotes apoptosis to prevent LUAD progression.

### Cancer-IgG Silencing Attenuates LUAD Resistance to Radiation Therapy

**Figure 3:**
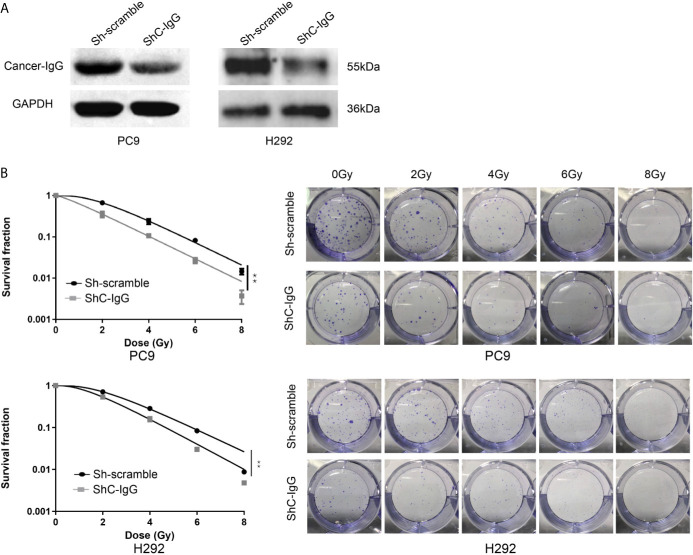
Cancer-IgG silencing attenuates lung adenocarcinoma resistance to radiation therapy. **(A)** Western blotting was used to test the efficiency of lentiviral interfering RNA in silencing cancer-IgG in PC9 and H292. **(B)** Colony-forming assay showed that the proliferation ability of PC9 and H292 cells at silencing cancer-IgG decreased after 0, 2, 4, 6, and 8 Gy of irradiation, and the single-hit multi-target model was fitted to the survival curve. **P < 0.01 (n=3). ShC-IgG: sh-cancer-IgG.

Radiation is one of the major therapeutic measures for LUAD. High-energy x-rays induce irreversible damage to the DNA of tumor cells, which cannot be repaired in time, and thus cause tumor cell death (24). To investigate whether cancer-IgG plays a role in the damage and repair process caused by radiation within LUAD cells and consequently affects radiosensitivity, the cancer-IgG in PC9 and H292 cells was first knocked down by lentivirus (**Figure 3A**). Then, the colony-forming test was conducted under irradiation with different radiation doses (0, 2, 4, 6, and 8 Gy). The number of colonies in the cancer-IgG knockdown group was significantly reduced. The cell survival curve was simulated by the single-hit multi-target model, which showed that the sensitivity of the cancer-IgG knockdown group to radiotherapy was enhanced (**Figure 3B**).

**Figure 4:**
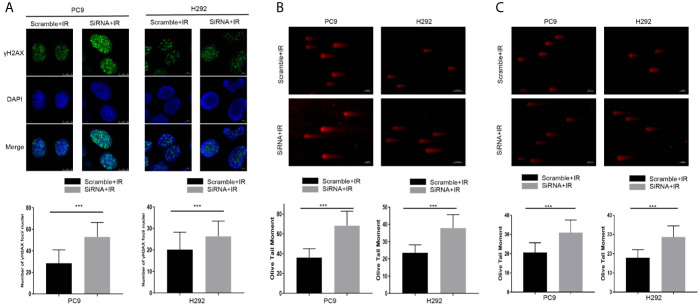
Cancer-IgG silencing attenuates lung adenocarcinoma resistance to radiation therapy. **(A)** Silencing cancer-IgG expression inhibited the DNA double-strand break repair ability of PC9 and H292 cells, and the number of γH2AX fluorescence foci in the nucleus increased. Count 50 cells in each group in the experiment. ***P < 0.001 (n=50). **(B, C)** The DNA double-strand break repair capacity was impaired in PC9 and H292 cells with cancer-IgG knockdown, and the Olive tail moment was extended. Count 50 cells in each group in the experiment. Scale bar, 50 μm. **(B)** Irradiation 4 Gy; **(C)** Irradiation 2 Gy. ***P < 0.001 (n=50).

Next, the γH2AX foci formation assay and neutral comet assay, considered critical for evaluating DNA damage process, were performed. As shown in **Figure 4A**, the number of γH2AX foci in nucleus increased 1 h after 4 Gy per fraction of irradiation in the cancer-IgG knockdown group, and the Olive tail moment was observed to have a significant increase 3 h after that irradiation (**Figure 4B**). In addition, under 2Gy irradiation, the results of the comet assay are consistent with the previous results (**Figure 4C**). The results indicated that cancer-IgG regulates the DSB damage repair process caused by radiotherapy, and cancer-IgG silencing attenuates LUAD resistance to radiation therapy.

### Cancer-IgG Mediates Radioresistance to LUAD *via* PI3K/AKT/DNA-PKcs Pathway

**Figure 5:**
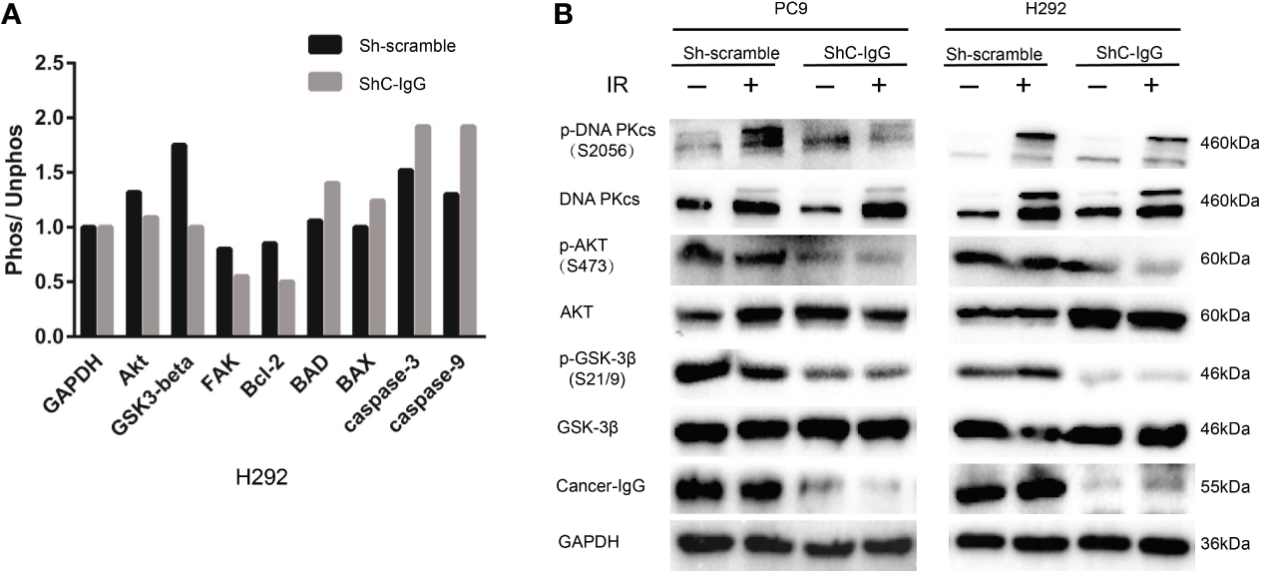
Cancer-IgG mediates radioresistance to LUAD *via* the PI3K/AKT/DNA-PKcs pathway. **(A)** An antibody phosphorylation chip was used to detect the phosphorylation changes in key proteins of the signaling pathway after cancer-IgG was silenced. **(B)** Western blot shows the changes in the expression of DNA-PKcs, p-DNA-PKcs, AKT, p-AKT GSK3β, and p-GSK3β of knockdown cancer-IgG under irradiated and nonirradiated conditions.

H292 sh-scramble and sh-cancer-IgG cells were irradiated with 6 Gy. After 6 h, the total protein was extracted for phosphorylation protein microarray analysis, which found that AKT and GSK3β protein phosphorylation ratios in the sh-cancer-IgG group were reduced and that Bcl-2, BAD, caspase 3, caspase 9, and BAX apoptosis-related proteins increased (**Figure 5A**). The results indicated that cancer-IgG knockdown under irradiation leads to inhibition of the PI3K/AKT pathway and increases apoptosis in LUAD cells. Furthermore, western blot analysis on PC9 and H292 cells showed that cancer-IgG knockdown under irradiation did not affect the expression of AKT, GSK3β, or DNA-PKcs proteins. However, the expression of p-AKT and p-GSK3β decreased; this result was consistent with that of the phosphorylation protein microarray analysis. p-DNA-PKcs, a core component in the NHEJ pathway for the DSB damage repair process, is phosphorylated under cellular stress and assembled into DNA-PK with Ku70 and Ku80 heterodimers to participate in the repair process of DSBs; they also cascade to amplify repair signals and recruit more sensor proteins to repair sites to promote repair (25). The results showed that expression of the p-DNA-PKcs protein in LUAD cells increased after radiation. Knockdown of cancer-IgG inhibited the expression of the p-DNA-PKcs protein (**Figure 5B**), suggesting that the damage repair ability of DSBs in LUAD cells decreased, and radiotherapy resistance was downregulated. These results suggest that cancer-IgG can mediate radiotherapy resistance in LUAD cells *via* the PI3K/AKT/DNA-PKcs pathway and that radiosensitivity can be promoted by knocking down the expression of cancer-IgG.

## Discussion

In this study, we presented evidence that the absence of cancer-IgG downregulates the phosphorylation of key proteins in the PI3K/AKT/DNA-PKcs pathway and thus attenuates the radiotherapy resistance of LUAD cells. We first found that cancer-IgG is expressed in the cell membrane and endoplasm of LUAD cells; the shorter OS and DFS of patients with positive cancer-IgG expression after radical postoperative chemoradiotherapy suggested an association between cancer-IgG and the poor prognoses as well as radiotherapy resistance. Cytological experiments provided confirmation of cancer-IgG expression in LUAD cells but not in normal epithelia cells and showed that, by knocking down cancer-IgG, the proliferation of LUAD cells can be inhibited, thus promoting apoptosis of tumor cells. In addition, after irradiation of LUAD cells with cancer-IgG knockdown, the cell proliferation ability was reduced even more, and the DSB repair ability was reduced. Mechanistic studies have shown that inhibiting cancer-IgG expression affects the PI3K/AKT/DNA-PKcs pathway to downregulate the expression of repair proteins, inhibit the DSB damage and repair of LUAD cells, and enhance the radiosensitivity. To our knowledge, this study is the first to demonstrate the role of cancer-IgG in radioresistance and suggest that cancer-IgG is a potential therapeutic target for LUAD radiation therapy.

Radiotherapy, considered a standard therapeutic measures for LUAD, plays a critical role in improving the survival and prognosis of patients but is greatly hindered by the existence of radioresistance of cancer cells (26). Radiation therapy combined with targeted DSB damage repair protein inhibitors has the potential for clinical application in patients with cancer; current drug development and clinical trials for targets such as DNA-PKcs, ATM/ATR (i.e., ataxia-telangiectasia mutated and Rad3-related protein kinases), and the MRN (i.e., MRE11-Rad50-NBS1) complex are underway and may, we hope, better explain the poor efficacy caused by radiotherapy resistance (27). Although cancer-IgG was discovered in cancer cells decades ago, previous studies mainly focused on the relationship between cancer-IgG and tumorigenesis. Wang and Gan (28) found that knockdown of cancer-IgG can regulate the PTP-BAS/Src/PDK1/AKT pathway and thus significantly promote cisplatin-induced apoptosis and inhibition of oral squamous cell proliferation, migration, and invasion. Qin et al. (9) found that, in prostate cancer, cancer-IgG staining was stronger in specimens at advanced clinical stages; androgen deprivation therapy for prostate cancer–induced cancer-IgG expression maintained stemness and facilitated cancer progression through mitogen-activated protein kinase/extracellular signal–regulated kinase and AKT in prostate cancer. In our study, staining of stage III LUAD slides suggested associations between cancer-IgG and tumor invasiveness, because expression approximately correlated with N staging and vascular tumor thrombus.

Building upon previous research, this study first explored the role of cancer-IgG in tumor radiotherapy and found that it is possible to mediate radiotherapy resistance by modulating the PI3K/AKT/DNA-PKcs signaling pathway. AKT, a serine/threonine kinase, is considered a core factor in the PI3K/AKT pathway, which functions mainly according to these four steps: survival factor induction, translocation to the cell membrane, phosphorylation, and activation of downstream effectors (29). The AKT protein is frequently deregulated in a variety of human cancers, leading to overactivation and promotion of tumor cell survival, proliferation, migration, metabolism, angiogenesis, and radiochemotherapy resistance by regulating the function of multiple downstream molecules (29–31). Moreover, AKT1 interacts with DNA-PKcs through its C-terminal domain to form a functional complex, stimulates the accumulation of DNA-PKcs at DSBs, promotes the activity of DNA-PKcs, and enhances radiation-induced DSB damage repair (32). PI3K/AKT inhibitors can significantly enhance radiosensitivity of cancer cells by targeting this pathway (33, 34). Many studies have claimed that AKT binds to DNA-PKcs and participates in promotion of binding to DNA damage sites and mediation of the trans/autophosphorylation of DNA-PKcs, thereby enhancing DSB damage repair in DNA (35–37). Our study demonstrated that the expressions of p-AKT and p-DNA-PKcs in cancer-IgG knockdown LUAD cells were decreased after radiation and that the repair ability of DSBs was decreased, as shown by results of the neutral comet assay and γH2AX immunofluorescence assay. These assays indicated that cancer-IgG can regulate the damage repair ability of DSBs in LUAD through the PI3K/AKT/DNA-PKcs pathway and upregulate radiotherapy resistance. Phosphorylation protein microarray analysis showed that, in addition to the decreased expression of damage repair proteins, apoptotic proteins of LUAD cells were increased, which may be due to the weakened repair ability and increased cell debris that enhance the mechanism of cell apoptosis.

To the best of our knowledge, this is the first study that explains the radiosensitization effect of cancer-IgG in LUAD. This study demonstrates that reducing the expression of cancer IgG effectively inhibits radiation-induced PI3K/AKT/DNA-PKcs signal transduction, resulting in impaired DSB damage repair ability, and leads to increased sensitivity to radiation treatments. The study provides new insights into cancer-IgG as a regulator of radiosensitivity in LUAD.

## Data Availability Statement

The raw data supporting the conclusions of this article will be made available by the authors, without undue reservation.

## Ethics Statement

The studies involving human participants were reviewed and approved by Medical Ethics Committee of Beijing Cancer Hospital. The patients/participants provided their written informed consent to participate in this study.

## Author Contributions

XY, GW, JY, RG, XX, CX, HW and RZ conducted the research. GZ designed the study. XQ contributed the essential reagents or tools. XY and RG wrote the paper. All authors contributed to the article and approved the submitted version. XY, GW, JY contributed equally.

## Funding

This study was supported by the grants from the National Key R&D Program of China (to GZ) (No.2018YFC1313202) and the China-Japan Friendship Hospital Scientific Research Start-up Funds (to GZ)(No.2016-RC-4).

## Conflict of Interest

The authors declare that the research was conducted in the absence of any commercial or financial relationships that could be construed as a potential conflict of interest.
